# A case report: Transcatheter aortic valve replacement via femoral artery for aortic regurgitation post-CARVAR

**DOI:** 10.3389/fcvm.2026.1908839

**Published:** 2026-08-18

**Authors:** Lin Li, Zhiming Zhou, Xiaoyu Wang, Lei Yin, Shenwei Zhang

**Affiliations:** 1 Department of Cardiology, The 7th People’s Hospital of Zheng Zhou, Zheng Zhou, Henan, China; 2 Department of Cardiovascular Surgery, Heart Transplantation Center, Henan Provincial Key Laboratory of Cardiac Remodeling and Transplantation, The 7th People’s Hospital of Zheng Zhou, Zheng Zhou, Henan, China; 3 Biotherapy Institute Henan Academy of Innovations in Medical Science, The 7th People’s Hospital of Zheng Zhou, Zheng Zhou, Henan, China

**Keywords:** AR-aortic regurgitation, capture device, CARAVR-comprehensive aortic root and valve repair, TAVR—transcatheter aortic valve replacement, ultra-high-level release

## Abstract

A 26-year-old male patient experienced recurrent severe aortic regurgitation accompanied by left ventricular enlargement and a left ventricular ejection fraction of 17.7% 8 years after undergoing comprehensive aortic root and valve repair (CARVAR). Given the high risks associated with redo sternotomy and the patient's explicit refusal of both redo sternotomy and heart transplantation, a multidisciplinary consultation led to the decision to perform transcatheter aortic valve replacement (TAVR) as a transitional treatment. The transvalvular procedure was performed with snare-assisted valve delivery devices and ultra-high release technique. Postoperative echocardiography demonstrated normal valve function without perivalvular leakage, with significant improvement in cardiac function compared to baseline. This case illustrates that TAVR can serve as an effective transitional therapeutic strategy for high-risk patients with recurrent aortic regurgitation following CARVAR.

## 1 Introduction

Aortic Regurgitation (AR) is one of the most common heart valve diseases (1, 2). For patients with isolated AR, Professor Song Myeong-Geun from South Korea has developed the comprehensive aortic root and valve repair (CARVAR) technique (3), aimed at improving cardiac function and reducing the long-term need for anticoagulant medications (3). By physiologically preserving the integrity of the aortic valve and root structures, CARVAR theoretically offers a longer functional lifespan. However, patients may still experience recurrent AR several years after the procedure (4). Given that CARVAR patients are generally younger at the time of their initial surgery, those who develop AR recurrence are often advised to replace the valve with a mechanical prosthesis to extend its lifespan. Nonetheless, the circumferential fixation of the sinotubular junction (STJ) during CARVAR and the subsequent significant left ventricular dilatation, combined with the adhesion issues from a prior sternotomy, pose significant challenges for re-operative redo sternotomy. Therefore, for patients at high risk for re-operative surgery, minimally invasive TAVR offers a novel therapeutic approach, providing an alternative treatment option for this patient population (5).

## 2 Case description

### 2.1 Preoperative clinical data

**Figure 1:**
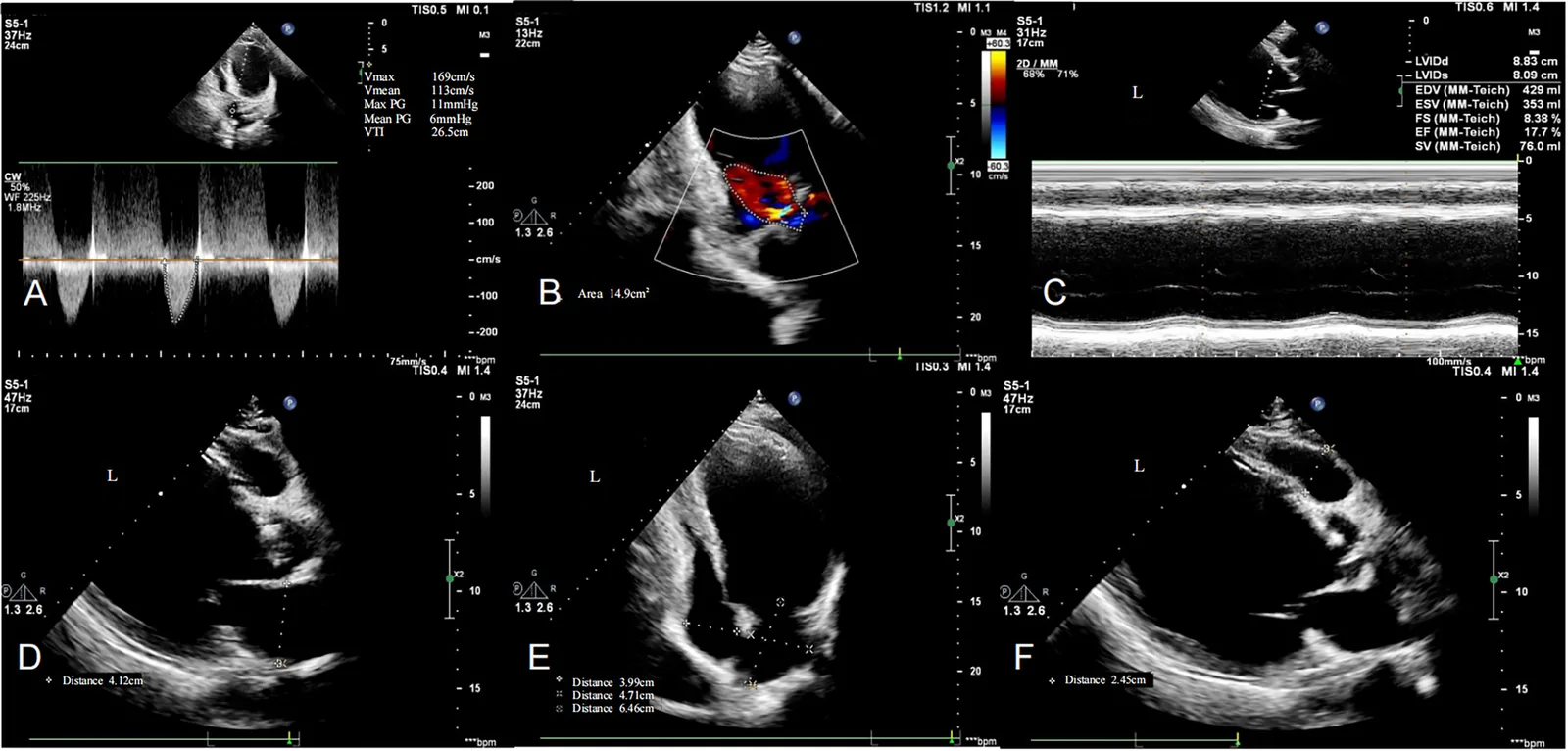
Preoperative echocardiography. **(A)** Aortic valve velocity 169 cm/s, peak pressure gradient 11 mmHg, mean pressure gradient 6 mmHg, velocity-time integral 26.5 cm; **(B)** Aortic regurgitation area 14.9 cm^2^; **(C)** Left ventricular end-diastolic diameter 88.3 mm, left ventricular end-systolic diameter 80.9 mm, ejection fraction 17.7%; **(D)** Left atrial anteroposterior diameter 41.2 mm; **(E)** Left atrial transverse diameter 47.1 mm, left atrial longitudinal diameter 64.6 mm, right atrial transverse diameter 39.9 mm; **(F)** Right ventricular anteroposterior diameter 24.5 mm.

**Figure 2:**
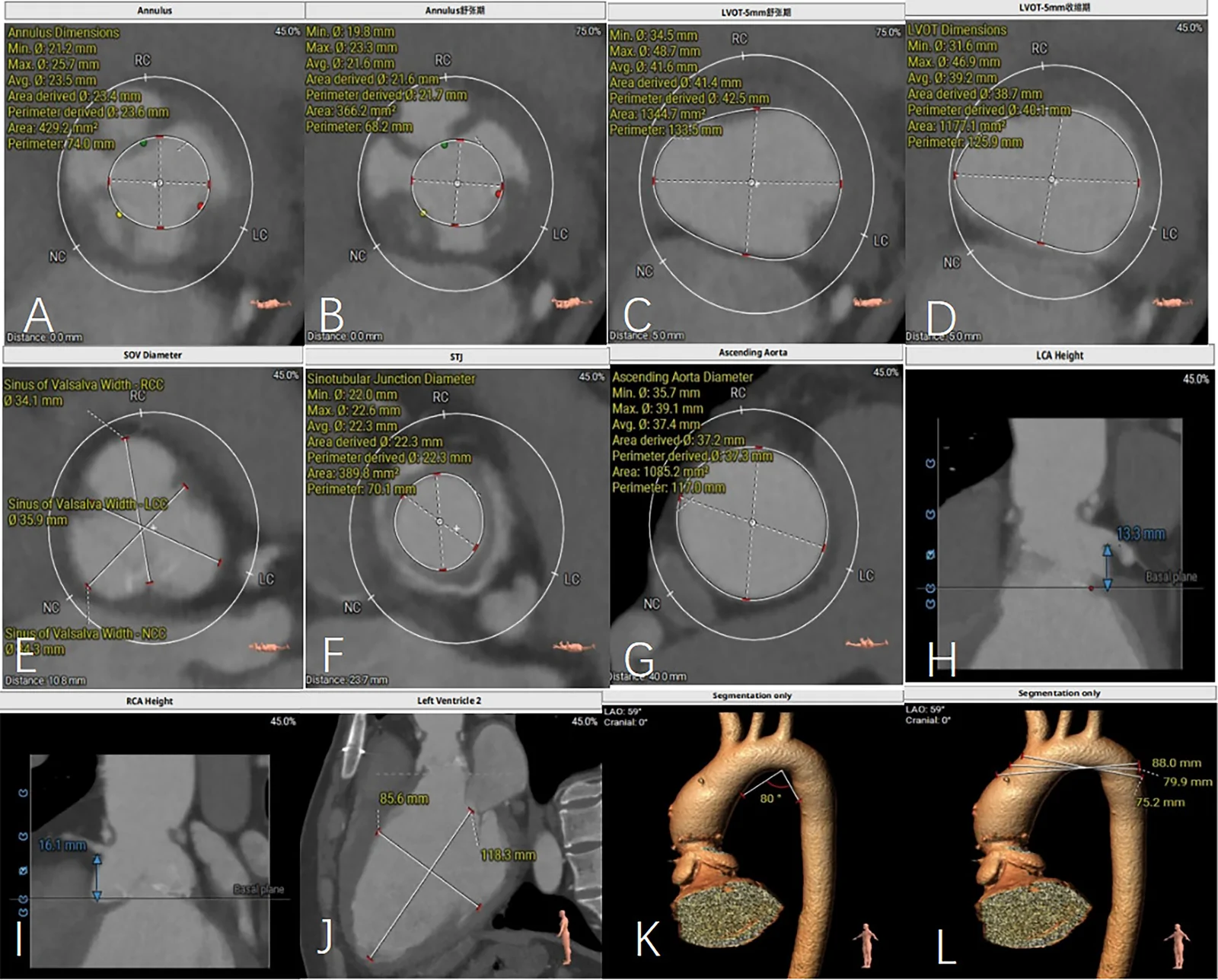
Preoperative CTA assessment. **(A)** Annular diameter during systole 29.9 mm; **(B)** Annular diameter during diastole 29.3 mm; **(C)** Diastolic diameter of the left ventricular outflow tract 42.5 mm; **(D)** Systolic diameter of the left ventricular outflow tract 40.1 mm; **(E)** Sinus of Valsalva diameters 34.1 mm, 35.9 mm, 34.3 mm; **(F)** Sinotubular junction diameter 22.3 mm; **(G)** Ascending aorta diameter 37.3 mm; **(H)** Height of the left coronary artery orifice 13.3 mm; **(I)** Height of the right coronary artery orifice 16.1 mm; **(J)** Left ventricular longitudinal diameter 118.3 mm, left ventricular transverse diameter 85.6 mm; **(K)** Aortic arch angle 80 degrees; **(L)** Aortic arch distance 88.0 mm.

A 26-year-old male patient initially reported symptoms of chest tightness and dyspnea following physical exertion 8 years ago. Echocardiography demonstrated a bicuspid aortic valve and severe AR. In 2017, the patient underwent CARVAR surgery under general anesthesia. Approximately 1 month prior to admission, the patient reported recurrence of chest tightness and dyspnea following physical exertion. The serum NT-proBNP level was 2,719.087 pg/mL. The patient's STS-PROM (Society of Thoracic Surgeons Predicted Risk of Mortality) score was calculated as 7.8%, consistent with guideline-defined high surgical risk for repeat cardiac surgery. Transthoracic echocardiography (TTE) revealed significant cardiac abnormalities, including left heart chamber enlargement, thickened aortic valve leaflets with severe regurgitation, diffuse hypokinesis of the left ventricular walls and the left ventricular ejection fraction (LVEF) was 17.7% (modified Simpson's method) (Figure 1). The patient declined both re-operation and heart transplantation. After multidisciplinary consultation, TAVR was proposed as a bridging therapy. Preoperative multi-slice CTA (Figure 2) yielded the following detailed anatomical measurements: (1) Enlarged left heart; (2) Mild thickening of the aorta and aortic valve without calcification. Based on the CTA results, the virtual annulus could not be determined, and TAVR valve release positioning was challenging.

### 2.2 Surgical procedure

Routine sterilization and draping were performed after connecting the electrocardiogram monitor. General anesthesia and sedation were administered. A temporary pacemaker electrode was inserted into the right internal jugular vein and positioned in the right ventricle as a reserve. The left femoral artery and femoral vein were punctured to insert a 6F arterial sheath as an auxiliary route, which would be used as a pathway for necessary extracorporeal membrane oxygenation (ECMO). The right femoral artery was punctured, with two Proglide sutures pre-embedded. The Taurus Elite 20F arterial sheath was slowly advanced along the stiff guide wire. With AL1 catheter support, a transvalvular guide wire was delivered into the left ventricle, exchanging the stiff guide wire.

**Figure 3:**
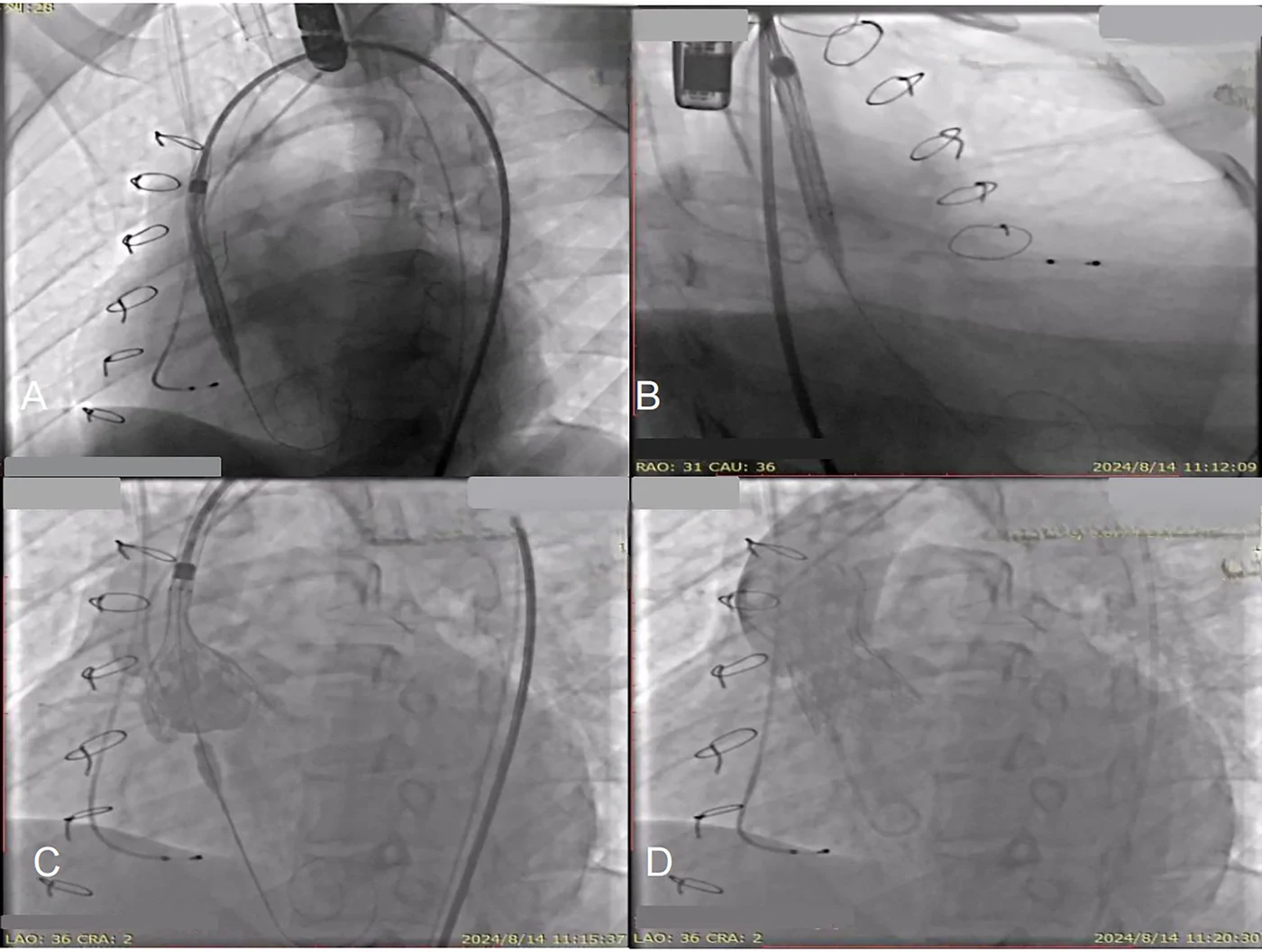
Intraoperative aortic root angiography. **(A)** The prosthetic valve is assisted across the valve using a snare; **(B)** The implantation is positioned 8–10 mm above the valve annulus, at a super-high starting position; **(C)** Post-deployment, the valve position is assessed at the final landing position; **(D)** angiography shows good position and morphology of the valve.

Due to distorted post-CARVAR aortic root anatomy lacking a calcified annular landing zone, traditional annular-based oversizing percentage calculation was not feasible. Valve sizing was guided by the 22.3 mm STJ diameter and 34.1–35.9 mm Valsalva sinus dimensions; the AV29 prosthesis was selected to generate sufficient radial force against thickened native leaflets for supra-annular fixation. Based on preoperative CT and echocardiography measurements, a Peijia AV29 prosthesis valve was selected and assembled onto the delivery system. With snare-assisted valve delivery, it was delivered to the annulus along the stiff guide wire. Overdrive pacing at 150 bpm was applied, and under the guidance of digital subtraction angiography (DSA) and ultrasound, the valve was released at a high position, gradually opening the valve stent to the working projection angle. Aortic root angiography showed a good position of the stent. After continuing to release the prosthesis valve, the catheter delivery system was retracted. We distinguish two spatial parameters to eliminate ambiguity: the primary supra-annular anchoring plane was set 8–10 mm above the native annulus to engage the narrow STJ ring and thickened leaflets; the valve inflow segment reached a final deployment depth of 0 mm on the non-coronary sinus side and 1 mm on the left coronary sinus side. Aortic root angiography showed no paravalvular leakage or obstruction of the left and right coronary arteries (Figure 3).

### 2.3 Postoperative follow-up

**Figure 4:**
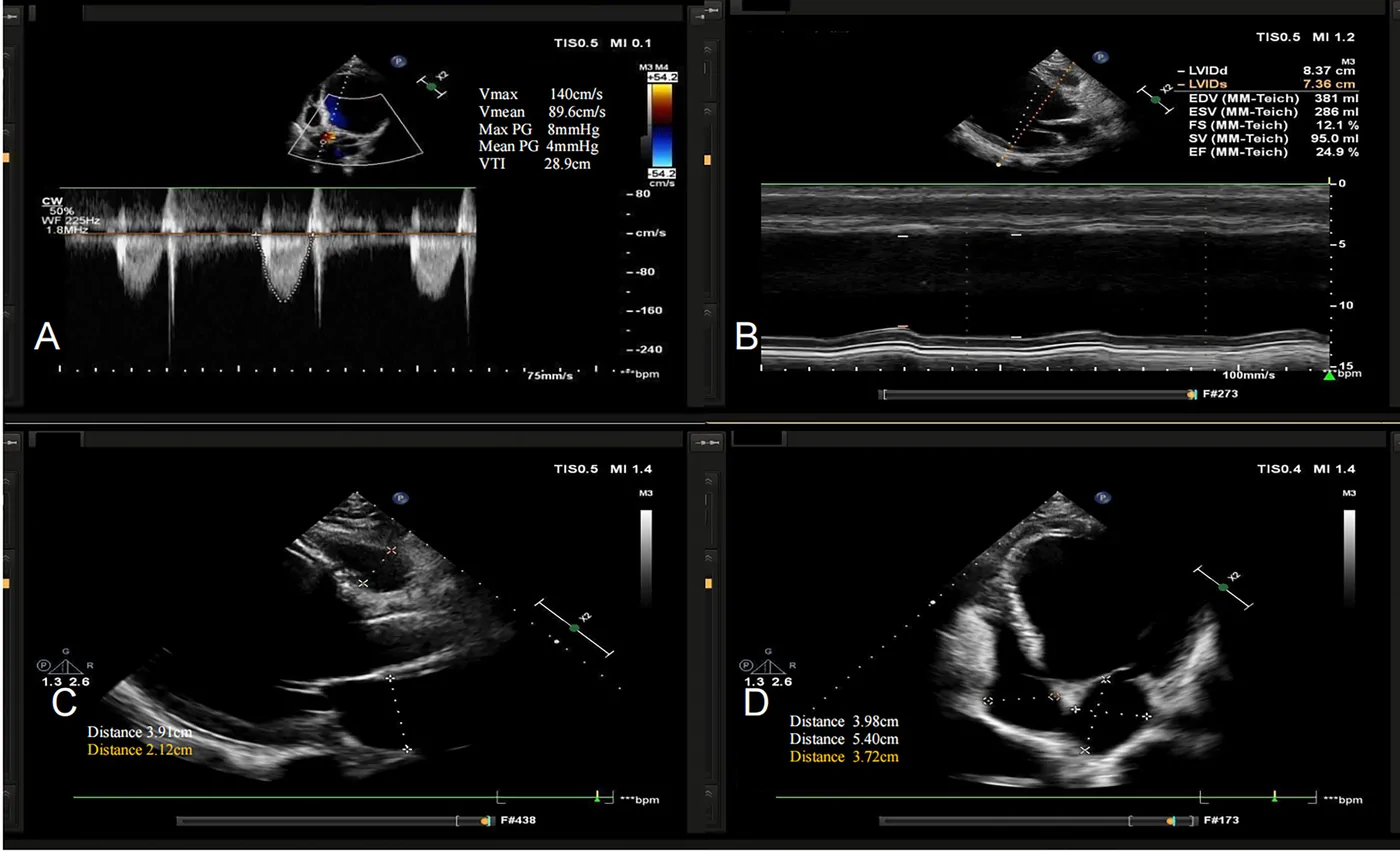
Three-month postoperative follow-Up echocardiography. **(A)** Aortic valve velocity 140 cm/s, peak gradient 8 mmHg, mean gradient 4 mmHg, velocity-time integral 28.9 cm; **(B)** Left ventricular end-diastolic diameter 83.7 mm; left ventricular end-systolic diameter 73.6 mm; ejection fraction 24.9%; **(C)** Left atrial anteroposterior diameter 39.1 mm, right ventricular anteroposterior diameter 21.2 mm; **(D)** Left atrial transverse diameter 39.8 mm, left atrial longitudinal diameter 54.0 mm, right atrial transverse diameter 37.2 mm.

Postoperatively, the patient continued to receive standard heart failure medication therapy. Three-Month Postoperative Follow-Up Echocardiography: The aortic valve leaflets exhibit free movement. The peak flow velocity at the valve orifice is 140 cm/s, with a peak pressure gradient of 8 mmHg and an average pressure gradient of 4 mmHg. No paravalvular leakage is observed. The left ventricular ejection fraction further increased (17.7% → 24.9%) (Figure 4).

## 3 Discussion

Current clinical experience with TAVR in patients following CARVAR remains limited, and the procedural challenges encountered in this population differ fundamentally from those in conventional aortic stenosis or regurgitation cases due to the surgically remodeled aortic root anatomy (6). The present case further illustrates this complexity, with four major intraoperative challenges identified. First, there was insufficient anchoring support in the left ventricular outflow tract: the STJ annuloplasty ring measured only 22.3 mm in diameter, while the left ventricular outflow tract (LVOT) diameter reached 40.1 mm, resulting in a lack of effective anchoring zones in the outflow tract and a substantial risk of valve migration. Valve fixation was achieved entirely through the thickened native leaflets and the constricted STJ segment, which represents a significant departure from conventional annular-based anchoring mechanisms. Second, the metallic STJ annuloplasty ring posed a potential obstacle to valve delivery, as the rigid ring could impede passage of the valve stent during crossing, potentially leading to delivery failure or aortic wall injury. Third, virtual annulus localization was extremely challenging, as the altered geometry and suspension of the repaired commissures following CARVAR fundamentally reshaped the virtual annular plane, markedly increasing intraoperative positioning difficulty. Fourth, the patient was at high risk of intraprocedural circulatory collapse due to severe left ventricular enlargement and profoundly reduced ejection fraction, rendering hemodynamic decompensation a major concern throughout the procedure.

Following comprehensive multimodality imaging assessment, the second-generation retrievable Taurus Elite self-expanding valve was selected for the procedure. Intraoperatively, a snare-assisted valve delivery technique was employed to avoid aortic arch injury and facilitate smooth transvalvular crossing. The valve was released at an ultra-high position 8–10 mm above the native annulus and adjusted to the standard working projection angle, with satisfactory valve position confirmed by multi-angle DSA and echocardiography. Two distinct anatomical positioning parameters should be clearly distinguished in this case: the supra-annular anchoring plane was set at 8–10 mm above the annulus, relying on the narrow STJ ring and thickened leaflets as the primary fixation zone, while the valve inflow segment reached a final deployment depth of 0 mm on the non-coronary sinus side and 1 mm on the left coronary sinus side. This ultra-high release strategy was not adopted arbitrarily but was the result of a deliberate integration of the patient's unique anatomical characteristics and clinical requirements. On one hand, the thickened native leaflets and the narrowed STJ ring constituted the only reliable anchoring structures in this distorted CARVAR root, and the valve stent's “waist” had to be precisely positioned at this level to achieve stable fixation. On the other hand, positioning the release plane 8–10 mm above the annulus substantially reduced mechanical contact between the stent frame and the membranous septum and His-Purkinje conduction system, thereby lowering the risk of new-onset high-grade atrioventricular block and permanent pacemaker implantation (7)—a consideration of particular importance for a 26-year-old patient with a life expectancy measured in decades. At the same time, this high-release strategy inevitably placed coronary protection at the center of the decision-making process. Preoperative multidetector CT quantitative assessment demonstrated that the left and right coronary ostial heights were 13.3 mm and 16.1 mm, respectively, both well above the planned valve landing zone, and the Valsalva sinuses were adequately wide (34.1–35.9 mm), indicating a low risk of acute coronary occlusion. Nevertheless, supra-annular implantation of a self-expanding valve may pose some challenge to future coronary angiography and interventions. One consideration in selecting the Taurus Elite was its supra-annular leaflet design, which preserves relatively generous neo-sinus space around the coronary ostia—compared to intra-annular balloon-expandable valves—thereby leaving more operating room for future coronary access. Regarding valve type selection, balloon-expandable valves were considered during preoperative planning but ultimately excluded for two reasons: these valves rely on radial force against a calcified annulus for stable fixation, whereas the present patient had no annular calcification and exhibited extreme LVOT dilatation, lacking a conventional landing zone altogether. In contrast, the self-expanding nature and retrievable design of the Taurus Elite not only permitted supra-annular positioning utilizing the STJ as an alternative anchoring point but also provided a “test-and-adjust” safety margin during deployment—a feature that proved particularly valuable in this complex anatomy with substantial variability and no standard landing zone. The Taurus Elite AV29 valve was successfully implanted with the expected procedural outcome achieved. Immediate post-deployment aortic root angiography revealed no paravalvular leakage and no obstruction of either coronary ostium. Three-month follow-up echocardiography demonstrated stable valve function, with left ventricular ejection fraction improving from 17.7% to 24.9% and a reduction in left ventricular chamber dimensions.

Comparison with the published case further highlights the unique value of the present report. In 2024, Gottschalk et al. first reported the use of a Medtronic Evolut FX valve via a standard, low intra-annular implantation approach in a post-CARVAR patient with preserved LVEF (65%) (8). Direct comparison between their case and ours reveals several critical distinctions that collectively define the novelty and clinical significance of this report. The most fundamental difference lies in the baseline hemodynamic status. Gottschalk's patient had preserved systolic function, whereas ours presented with end-stage heart failure characterized by a critically depressed LVEF of 17.7% and severe left ventricular dilatation (LVEDD 88.3 mm)—a phenotype never previously documented in the literature on TAVR post-CARVAR. This extreme clinical scenario demanded not only technically successful valve implantation but also a procedure that could serve as a life-saving “bridge to recovery,” a therapeutic role that had not been validated in this population until now. Compounding this hemodynamic challenge was the substantially more hostile anatomy in our case: the STJ diameter was only 22.3 mm, far smaller than the 25.5 mm reported in the comparator case, forcing exclusive reliance on the STJ and thickened native leaflets as the sole anchoring mechanism—a technical difficulty that was not addressed in the prior literature. To overcome these combined challenges, we adopted a procedural strategy diametrically opposite to the deep intra-annular implantation used previously: a customized ultra-high release technique (8–10 mm above the annulus) with the Peijia Taurus Elite self-expanding valve. This tactical choice yielded a clinically meaningful advantage—the avoidance of permanent pacemaker implantation, whereas the prior case required postoperative pacing—which is of particular importance for our 26-year-old patient, far younger than the 76-year-old subject in the published report, raising distinct long-term concerns regarding valve durability and future coronary access that are not pertinent to the elderly population. Finally, our 3-month follow-up data provide objective echocardiographic evidence of progressive left ventricular reverse remodeling (LVEF improved from 17.7% to 24.9% with a trend toward dimensional reduction), information that was absent from the short-term follow-up of the previous case. Collectively, these distinctions establish that our report is not a mere repetition of prior work but a meaningful extension of the evidence base, demonstrating TAVR feasibility in a substantially sicker patient population and offering an alternative technical strategy with distinct advantages in conduction preservation and mid-term cardiac recovery.

This report, as a single-case study, has inherent limitations. First, only 3-month mid-term follow-up data are available, which are insufficient to assess long-term valve durability or late coronary-related complications in a young patient. Second, as a single-center, single-case experience, its generalizability is limited; large-scale, multicenter prospective cohort studies are needed to validate the safety and efficacy of this ultra-high release technique across various post-CARVAR anatomical variants. Third, there is currently no established standard for long-term management of TAVR as a bridging therapy; whether subsequent elective redo sternotomy or repeat TAVR is warranted should be determined individually based on long-term valve function and the extent of ventricular remodeling recovery. In summary, this case confirms that TAVR is a feasible and effective bridging therapy for high-risk patients with recurrent aortic regurgitation following CARVAR who are unable to tolerate redo sternotomy. Compared with conventional open-heart surgery, TAVR avoids the risks associated with cardiopulmonary bypass and repeat sternotomy, making it particularly suitable for patients with severe left ventricular dysfunction and high surgical risk. Rigorous preoperative CTA evaluation and intraoperative multimodal imaging guidance are essential for procedural success. The ultra-high release technique can reduce the risk of valve migration due to insufficient outflow tract anchoring, while the retrievable valve system provides intraoperative adjustability and an additional safety margin.

## 4 Conclusion

TAVR serves as a viable therapeutic option for patients suffering from recurrent aortic regurgitation after CARVAR repair. Our results highlight the characteristic anatomical remodeling of the aortic root and virtual annulus induced by CARVAR surgery, and stress that thorough multimodal preoperative imaging evaluation together with personalized, detailed procedural planning are indispensable to reduce perioperative hemodynamic complications and intraoperative technical risks. The combination of snare-assisted valve delivery and customized ultra-high deployment TAVR implemented in this work constitutes an innovative personalized treatment paradigm, which requires further validation via prospective clinical research.

## Data Availability

The original contributions presented in the study are included in the article/Supplementary Material, further inquiries can be directed to the corresponding authors.
